# Association between childhood psychiatric disorders and psychotic experiences in adolescence: A population-based longitudinal study

**DOI:** 10.1016/j.comppsych.2016.05.004

**Published:** 2016-08

**Authors:** Caroline Siebald, Golam M. Khandaker, Stanley Zammit, Glyn Lewis, Peter B. Jones

**Affiliations:** a Department of Psychiatry, University of Cambridge, UK; b Cambridgeshire and Peterborough NHS Foundation Trust, Cambridge, UK; c Centre for Mental Health, Addiction and Suicide Research, School of Social and Community Medicine, University of Bristol, UK; d Institute of Psychological Medicine and Clinical Neurosciences, MRC Centre for Neuropsychiatric Genetics and Genomics, Cardiff University, UK; e Division of Psychiatry, University College London, London, UK

## Abstract

**Background:**

Adolescent psychotic experiences (PEs) are common, and are associated with both psychotic and non-psychotic illnesses. In order to examine psychopathological and cognitive antecedents of adolescent PEs, we have conducted a longitudinal study of common childhood psychiatric disorders and subsequent adolescent PEs in the population-based prospective ALSPAC birth cohort.

**Method:**

Depression, anxiety, attention deficit hyperactivity disorder, oppositional defiant or conduct disorder, and pervasive developmental disorder were diagnosed according to DSM-IV criteria in 8253 participants at age 8 years. IQ was assessed by WISC-III also at 8 years. PEs, depressive and anxiety symptoms were assessed at 13 years. Logistic regression calculated odds ratio (OR) for PEs at 13 years associated with psychiatric disorders at 8 years. Linear regression calculated mean difference in IQ between groups with and without psychiatric disorder. Mediating effects of IQ, mood and anxiety symptoms on the psychiatric disorder-PEs relationship were examined.

**Results:**

In total, 599 children were assessed to have a DSM-IV psychiatric disorder at 8 years (7.2%). These children compared with those without any psychiatric disorder performed worse on all measures of IQ; adjusted mean difference in total IQ − 6.17 (95% CI, − 7.86, − 4.48). Childhood psychiatric disorders were associated with PEs subsequently in adolescence; adjusted OR 1.96 (95% CI, 1.47–2.68). The association between psychiatric disorder and subsequent PEs was partly mediated by, independently, IQ deficit at 8 years and depressive and anxiety symptoms at 13 years.

**Conclusions:**

The findings indicate that adolescent PEs are associated with general cognitive ability and past and present psychopathological factors.

## 1 Introduction

Psychotic experiences (PEs) are common in the general population [1], especially during childhood and adolescence [2]. Early-life PEs are associated with increased risk of psychosis in adulthood [3], [4] as well as a number of established risk factors for schizophrenia including family history of psychosis, cannabis use, maltreatment, advanced paternal age, IQ deficit, prenatal and childhood infection, and other immunological factors [5], [6], [7], [8], [9], [10], [11], [12], [13], [14], [15]. However, recent studies suggest that PEs can no longer be regarded as having predictive specificity for psychotic disorders subsequently in adulthood [16], [17]. PEs are associated with a range of non-psychotic common mental disorders both cross-sectionally and longitudinally. PEs in adolescence or adulthood are associated with concurrent anxiety, depression, obsessive compulsive disorder, borderline personality disorder, poor functioning, self-harm and suicidal behavior [18], [19], [20], [21], [22], [23]. These findings are consistent with statistical modeling of the underlying structure of psychiatric symptoms in two birth cohorts which reveal that psychotic phenomena co-occur with depression and anxiety [24], [25], and may be a marker of the severity of mental ill health in a single, unitary dimension of common mental distress in young people [25].

Longitudinal studies suggest that PEs are associated with a range of psychiatric disorders subsequently in adulthood including obsessive compulsive disorder, social phobia, dysthymia, bipolar disorder, post-traumatic stress disorder, and suicidality [16], [17]. Persistent childhood PEs are associated with subsequent internalizing and externalizing psychopathology in the general population [26]. Reports from the Avon Longitudinal Study of Parents and Children (ALSPAC) birth cohort [27], [28], including our own [5], have shown an association between childhood neurodevelopmental disorders and risk of PEs in early-adolescence, which is partly mediated by childhood IQ deficit [5]. However, to our knowledge no population-based longitudinal study has examined the relationship between common mental disorders of childhood diagnosed according to clinical criteria and risk of PEs subsequently in adolescence. Building on our previous work we have examined psychopathological and cognitive antecedents of adolescent PEs using a broader range of childhood psychiatric disorders as predictors of subsequent PEs in early-adolescence in the ALSPAC birth cohort. We hypothesized that psychiatric disorders at age 8 years would be associated with increased risk of PEs at age 13 years. The use of clinical diagnoses of depression, anxiety, oppositional defiant/conduct disorder, attention deficit hyperactivity disorder and pervasive developmental disorder at age 8 years defined according to DSM-IV criteria as predictors of PEs at age 13 years – as opposed to parent-reported neurodevelopmental disorders at age 10 years used in the previous study [5] – makes the current analysis unique. We have also examined mediating effects of IQ at 8 years, and depressive and anxiety symptoms at 13 years on the relationship between psychiatric disorder at 8 years and PEs at 13 years.

## 2 Method

### 2.1 Sample

The ALSPAC birth cohort is based on all pregnant women resident in the county of Avon, a geographically defined region in the southwest of England, with expected dates of delivery between April 1991 and December 1992 (http://www.bristol.ac.uk/alspac/). The initial ALSPAC cohort consisted of 14,062 live births and 13,988 infants still alive at 12 months [29], [30]. Avon included both urban and rural areas, and the population was broadly representative of all children in the UK. The parents completed regular postal questionnaires about all aspects of their child's health and development since birth. Since the age of 7 years the children attended an annual assessment clinic during which they participated in a range of face-to-face interviews and physical tests. The current study is based on 8253 individuals who were assessed for psychiatric disorders at age 8 years. The numbers of individuals with data on IQ at 8 years and psychiatric symptoms at 13 years vary as these tests were completed by different numbers of people.

Ethical approval for the study was obtained from ALSPAC Ethics and Law Committee and the Local Research Ethics Committees.

### 2.2 Assessment of psychiatric disorders at 8 years

Psychiatric disorders were assessed at age 8 years using the parent version of the Development and Well-Being Assessment (DAWBA) [31] and were coded according to DSM-IV criteria by two experienced psychiatrists [32]. The DAWBA consists of a package of questionnaires that are aimed at establishing the presence of relatively common emotional, behavioral, and hyperactivity disorders in children. It has been validated for use in epidemiological studies in the UK, where it has been used for the child and adolescent mental health survey [33], and internationally [34], [35].

A postal questionnaire containing the parent-version of the DAWBA was sent to mothers when the study child was on average about 8 years old. The parent-completed DAWBA-questionnaire constituted the primary source of information for the diagnosis of psychiatric disorders. Where available, complementary sources of information on a child's emotional and behavioral characteristics were consulted, which included: (i) the teacher version of the DAWBA that addressed potential hyperactivity and conduct disorder at 8 years; (ii) the teacher version of the Strengths and Difficulties Questionnaire (SDQ) at 8 years [36]; (iii) parent completed SDQ at 7 years; (iv) basic reading and spelling competency of the child at age 7 years; and (v) IQ at age 8 years using the Wechsler Intelligence Scale for Children (WISC III, 3rd UK edition) [37]. Psychiatric diagnoses were thus based on a wealth of developmental data gathered from different sources. Diagnoses were made for attention deficit hyperactivity disorder (ADHD), oppositional or conduct disorder (OpCD), pervasive developmental disorder (PDD), anxiety disorder, and depressive disorder. The presence of any DSM-IV psychiatric disorder at 8 years, coded as a single binary variable, was used as the main predictor. Those without a diagnosis were included in the comparison group. Additional analyses were carried out using individual diagnostic categories as predictors, which included all participants without the particular diagnosis in the comparison group. For example, children with depression who did not have ADHD were included in the comparison group for the analyses of ADHD, and so on.

### 2.3 Assessment of IQ at 8 years

Full scale, verbal, and performance IQ were measured by the WISC III, 3rd UK edition [37]. A shortened version of the test was applied by trained psychologists, whereby alternate items (always starting with item number 1 in the standard form) were used for all ten subtests with the exception of the coding subtest which was administered in its standard form. Use of the shortened version reduced the length of assessment so the children were less likely to tire. This approach has been successfully used in other studies [5], [38], [39]. IQ data obtained using this method have shown robust correlations with neurodevelopmental disorders, and other concurrent neurocognitive measures such as working memory, short-term memory, and sociodemographic factors such as social class [5].

### 2.4 Assessment of psychotic experiences at 13 years

Psychotic experiences (PEs) were identified through the face-to-face, semi-structured Psychosis-Like Symptom Interview (PLIKSi) conducted by trained psychology graduates in assessment clinics. The PLIKSi has good inter-rater reliability (kappa = 0.7) [9]. It comprised 12 ‘core’ questions derived from the Diagnostic Interview Schedule for Children-IV (DISC-IV) [40], and coded according to the rating rules for the Schedules for Clinical Assessment in Neuropsychiatry version 2.0 (SCAN 2.0) [41]. It included key symptoms covering the three main domains of positive psychotic symptoms: hallucinations (visual and auditory); delusions (spied on, persecution, thought being read, reference, control, grandiose ability, and other unspecified); and experiences of thought interference (insertion, withdrawal, and broadcasting). The observer based rating for the presence of any PEs (suspected or definite) in the past six months was used as the outcome. The group with PEs was compared with the rest of the cohort.

### 2.5 Assessment for depressive and anxiety symptoms at 13 years

Symptoms of depression and anxiety were measured at age 13 years using the short version of the mood and feelings questionnaire (MFQ) completed by the participants [36]. The short MFQ is a validated tool widely used in epidemiological studies [42], [43]. It includes 13 items covering core symptoms of depression and anxiety experienced in the past two weeks. Each item is scored zero (not true), one (sometimes true) or two (true) giving a total score of 0–26.

### 2.6 Statistical analysis

Baseline characteristics between the groups with and without any psychiatric disorder at 8 years were compared by independent sample t-test (continuous data) and chi-squared test (categorical data). Linear regression calculated the mean difference (95% CI) in IQ scores at 8 years between those with and without any psychiatric disorder. Binary logistic regression calculated the odds ratio (OR) for PEs at 13 years in the group with any psychiatric disorder at 8 years compared with those without. All regression models were controlled for age at assessment, sex, mother's social class, and highest educational level.

Mediating effect of IQ at 8 years on the association between psychiatric disorders at 8 years and PEs at 13 years was examined two ways. First, separate regression models assessed the associations between: (1) exposure (any psychiatric disorder) and outcome (PEs); (2) exposure and mediator (total IQ score); (3) mediator and outcome; and (4) exposure and outcome controlling for mediator. We expected, in the final step, that the exposure–outcome relationship would be attenuated (partial mediation) or eliminated (complete mediation). In case of partial mediation, the extent to which the psychiatric disorder-PEs estimate was attenuated after inclusion of total IQ (the mediator) was calculated. Second, mediation analyses were conducted using the STATA package ‘paramed’. It calculated the controlled direct effect (i.e. the effect of any psychiatric disorder (exposure) on the risk of PEs (outcome) after adjusting for IQ); natural direct effect (i.e. the effect on the risk of PEs of a change in IQ among unexposed individuals compared with exposed individuals); and total effect (the product of controlled direct effect and natural direct effect). Mediating effect of total MFQ score at 13 years on the association between psychiatric disorders at 8 years and subsequent PEs at 13 years was examined using the same approach for IQ described above. The ‘paramed’ approach was omitted as MFQ scores were not normally distributed. Instead, the association between psychiatric disorders at 8 years and PEs at 13 years was examined by stratifying the sample according to tertiles of MFQ score.

## 3 Results

### 3.1 Psychiatric disorders at 8 years and baseline characteristics

**Fig. 1:**
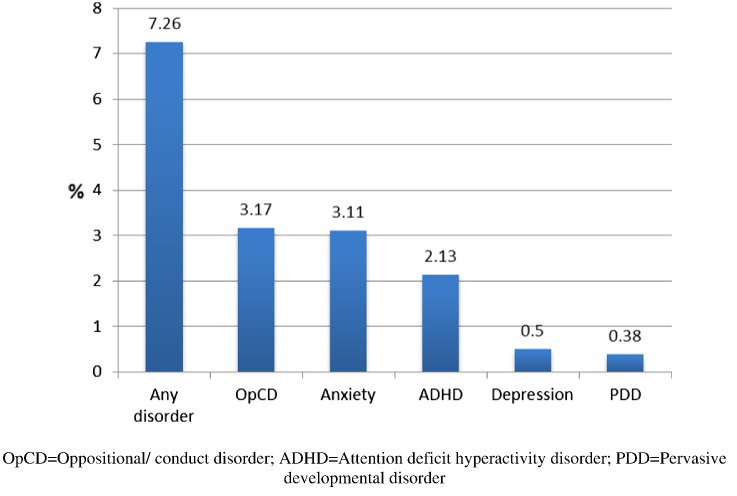
Prevalence of common psychiatric disorders (DSM-IV) at 8 years in the ALSPAC birth cohort.

At 8 years, 599 out of total 8253 children (7.2%) were assessed to have a DSM-IV psychiatric disorder (depression, anxiety, oppositional defiant/conduct disorder, ADHD, PDD). Out of these, 465 children had one (5.6%), 136 children had two (1.3%), and 28 children had three or more psychiatric disorders (0.3%). Oppositional/conduct disorder was the most common (Fig. 1). Psychiatric disorders were more common in boys (Table 1).

### 3.2 Association with IQ at 8 years

Compared with children with no psychiatric disorder, children with a psychiatric disorder as a group performed worse on all measures of IQ at 8 years (Table 2). The results remained similar after adjusting for a number of potential confounders. We also examined total IQ at 8 years separately in five specific psychiatric disorders. Compared with the rest of the cohort, mean total IQ was lower in all disorders except depression. The largest deficit in total IQ was observed for pervasive developmental disorder.

### 3.3 Association with psychotic experiences at 13 years

Data on psychiatric disorders at 8 years and the outcome of PEs at 13 years were available for 5528 individuals. In the group with psychiatric disorders 75 developed PEs (20.8%), while in the group with no psychiatric disorder 673 developed PEs (13.0%). There was nearly two-fold increased risk of PEs at 13 years for individuals with any psychiatric disorder at 8 years compared with those without. The association persisted after adjusting for potential confounders (Table 3). Separate analyses using specific psychiatric disorders showed that all childhood disorders were individually associated with PEs in adolescence; the highest OR was observed for pervasive developmental disorder (Table 3).

### 3.4 Effect of IQ at 8 years

Step by step regression analyses showed that about a fifth (21%) of the association between psychiatric disorders at 8 years and PEs at 13 years could be accounted for by deficit in total IQ at 8 years among children with psychiatric disorders. The association between psychiatric disorders at 8 years and PEs at 13 years (OR 1.76; 95% CI, 1.35–2.30) was attenuated after including total IQ score at 8 years as a predictor (OR 1.56; 95% CI, 1.16–2.10). This finding was consistent with additional mediation analyses, which revealed that a small but significant part of the relationship between psychiatric disorders and subsequent PEs could be accounted for by IQ. The overall adjusted OR for PEs at 13 years for psychiatric disorders at 8 years was 1.66 (95% CI, 1.19–2.33) after taking into account sociodemographic factors as well as total MFQ score at 13 years (i.e. total effect), which was composed in part by IQ deficit among children with a psychiatric disorder; adjusted OR 1.05 (95% CI, 1.01–1.10) (Table 4).

### 3.5 Effect of MFQ score at 13 years

Step by step regression analyses showed that about 13% of the association between psychiatric disorders at 8 years and PEs at 13 years could be accounted for by higher MFQ scores at 13 years among children with psychiatric disorders. The association between psychiatric disorders at 8 years and PEs at 13 years (OR 1.76; 95% CI, 1.35–2.30) was attenuated after including total MFQ score at 13 years as a predictor (OR 1.63; 95% CI, 1.23–2.17). Further examination of the association between psychiatric disorders at 8 years and PEs at 13 years was carried out dividing the sample into thirds according to tertiles of MFQ score. It showed that the association between psychiatric disorders at 8 years and PEs at 13 years was present only in participants in the top third of MFQ scores (OR 1.65; 95% CI, 1.14–2.40). The ORs for those in the bottom (1.74; 95% CI, 0.95–3.16) and middle (1.53; 95% CI, 0.82–2.84) thirds of MFQ scores were not statistically significant.

## 4 Discussion

The findings suggest that childhood psychiatric disorders diagnosed according to DSM-IV criteria at 8 years are associated with increased risk of psychotic experiences at 13 years, which persists after controlling for potential confounders such as age, sex, ethnicity, maternal social class and educational level. IQ at 8 years and depressive and anxiety symptoms at 13 years both independently mediated part of the relationship between childhood psychiatric disorders and subsequent PEs in adolescence. Although all childhood psychiatric disorders were associated with adolescent PEs, the highest OR was observed for PDD. Over half of the children with PDD developed PEs at 13 years compared with about an eighth in the comparison group. However, multivariable regression analyses for PDD lacked statistical power as reflected by wide confidence interval around the ORs because only a small number of participants met the criteria for this diagnosis. As expected, this group also had the highest deficit in IQ. These findings are consistent with the neurodevelopmental hypothesis of schizophrenia which posits abnormal neurodevelopment as a cause of the illness [44], [45]. Population-based longitudinal studies reporting impaired motor, cognitive, language and social development during infancy, childhood and adolescence in future cases of schizophrenia support the neurodevelopmental hypothesis [46], [47], [48], [49], [50], [51], [52], [53]. Meta-analysis of population-based longitudinal studies has confirmed a premorbid IQ deficit of about half a standard deviation in future cases of schizophrenia compared with healthy controls [54]. The findings are also consistent with three previous studies from the ALSPAC cohort reporting about two-fold increased risk of PEs in adolescence for autism, dyslexia and other neurodevelopmental disorders in childhood [5], [27], [28], although lack of an association between autistic traits and PEs has also been reported [55]. The use of clinical diagnosis of PDD as defined by DSM-IV criteria as exposure, as opposed to neurodevelopmental symptoms [27], [28] or parent-reported disorder [5] used in the previous studies, could explain a larger effect observed in the current study.

Looking at the underlying mechanism for PEs, the study shows cognitive deficit (as measured by IQ) and increased depressive and anxiety symptoms (as measured by MFQ score) both independently, in part explain the risk of adolescent PEs associated with past psychopathology. The findings are consistent with the idea that adolescent PEs have a cognitive facet to their origin. Indeed, adolescent PEs assessed at 13 years have been previously reported to be associated with lower childhood IQ at 8 years in the ALSPAC cohort [9]. Similarly, PEs at 12 years have been reported to be associated with lower IQ at 5 years in another cohort [10]. The findings are also consistent with previous population-based studies showing an association between PEs and other psychiatric symptoms such as depression and anxiety. Population-based studies have previously shown that adolescent PEs may be a marker of multiple concurrent depressive or anxiety disorders (i.e. severity of mental ill-health) or of the likelihood of clinically concerning behaviors such as suicidal thoughts or self-harm [18], [19], [20], [21]. Moreover, item response theory analysis of the underlying structure of psychiatric symptoms in the ALSPAC birth cohort has revealed that psychotic phenomena co-occur with depression and anxiety where they represent severity of mental ill-health in a single, unitary dimension of common mental distress in young people [25]. Thus, the current study supports the previous findings by showing that concurrent depressive and anxiety symptoms partly account for adolescent PEs.

The use of a general population-based birth cohort, large sample size, objective assessments for IQ, and PEs are particular strengths of this study. Diagnoses of psychiatric disorders at 8 years were based on primarily parent completed DAWBA questionnaire. Although where available complementary sources of information on a child's emotional and behavioral characteristics were consulted (see Methods) the use of parent reported data as opposed to direct assessment to establish a diagnosis is a limitation of this study. We did not have data on family psychiatric history and substance use to examine whether these variables confound the association between childhood psychiatric disorder and subsequent PE in early-adolescence, which is a potential limitation of this study. Another limitation is missing data. A third of participants assessed for psychiatric diagnoses at 8 years did not attend assessment for PEs at 13 years. Presence of a psychiatric disorder in childhood was associated with missing data for PEs at follow-up. If participants with psychiatric disorder at 8 years who did not develop PEs at 13 years were more likely to be missing at follow-up this would lead to a spurious over estimation of the association between childhood psychiatric disorder and subsequent PEs. However, this is unlikely. Missing data were also associated with lower maternal education and lower maternal socio-economic status. In the ALSPAC cohort both of these factors are associated with higher PEs, lower IQ, and adverse health and social outcomes. PEs were not assessed at baseline. Although we cannot be certain that it is likely that PEs at 13 years were new onset phenomena in majority of subjects since baseline assessment at age 8 years, this is because early-life PEs are transitory phenomena in most children; a 6-year follow-up of the ALSPAC birth cohort suggests that about 80% of subjects with PEs in early-adolescence do not have PEs at age 18 years [3].

To our knowledge, this is one of the first population-based longitudinal studies of common childhood psychiatric disorders and subsequent PEs in early-adolescence. The findings implicate past and present psychopathological factors as well as general cognitive ability in the origin of adolescent PEs. In the future, studies should use repeated assessment of PEs over the early life course in order to elucidate factors associated with persistence and remission of these symptoms. Individuals with childhood psychiatric disorders are known to be at higher risk of developing psychotic and non-psychotic illnesses during adult life. In the future studies should also examine whether adolescent PEs can account for the association between childhood and adult psychiatric disorders.

## Declaration of interest

None of the authors has any conflicts of interest to declare. Prof Jones received an honorarium that he donated to his department from Roche for taking part in an advisory board to advise on education about schizophrenia to psychiatrists. He directs the National Institute for Health Research Collaborations for Leadership in Applied Health Research and Care for Cambridgeshire and Peterborough (CLAHRC-CP) of which this work forms part.

## Figures and Tables

**Table 1:** Baseline characteristics of individuals with psychiatric disorders at 8 years.

Characteristic	Psychiatric disorder	No disorder	P-value^a^
Total No.	599	7654	-
Male sex, no. (%)	413 (68.9)	3832 (50.1)	< 0.001
Age at assessment, mean (SD), years	7.69 (0.15)	7.68 (0.14)	0.400
British white ethnicity, no. (%)	565 (98.6)	7253 (98.2)	0.819
Mother's social class, no. (%)			0.017
I (professional)	20 (4.20)	462 (7.26)	
II (intermediate)	154 (32.3)	2177 (34.2)
III nm (skilled, non-manual)	208 (43.8)	2705 (42.5)
III m (skilled, manual)	33 (6.93)	422 (6.63)
IV (partly skilled)	47 (9.87)	501 (7.87)
V (unskilled)	14 (2.94)	100 (1.57)
Maternal highest education, no. (%)			0.643
Secondary school	93 (16.2)	1053 (14.2)
Vocational	52 (9.10)	660 (8.90)
O level	196 (34.2)	2607 (35.2)
A level	149 (26.0)	1903 (25.6)
Degree	83 (14.5)	1189 (16.04)

a Independent sample t-test for continuous data (age), chi-squared test for proportions (sex, ethnicity, maternal social class, and education).

**Table 2:** IQ at 8 years in children with and without psychiatric disorders.

Psychiatric disorder/IQ	Psychiatric disorder		No disorder		Mean difference (95% CI)	
	No.	Mean (SD)	No.	Mean (SD)	Unadjusted	Adjusted^a^
*Any psychiatric disorder*						
Total IQ	400	98.50 (18.63)	5665	105.57 (16.03)	− 7.06 (− 8.71, − 5.42)	− 6.17 (− 7.86, − 4.48)
Verbal IQ	402	101.96 (19.10)	5691	108.44 (16.40)	− 6.48 (− 8.18, − 4.80)	− 5.94 (− 7.66, − 4.21)
Performance IQ	401	94.82 (18.75)	5683	100.79 (16.76)	− 5.97 (− 7.68, − 4.26)	− 4.85 (− 6.69, − 3.01)

*Individual disorder* (*total IQ*)						
Oppositional/conduct	175	100.22 (17.42)	5880	105.28 (16.22)	− 5.07 (− 7.51, − 2.62)	− 3.03 (− 6.08, − 0.98)
Anxiety	174	100.73 (17.29)	5891	105.23 (16.26)	− 4.50 (− 6.95, − 2.04)	− 4.13 (− 6.63, − 1.64)
ADHD	116	93.84 (21.34)	5939	105.36 (16.08)	− 11.51 (− 14.49, − 8.54)	− 9.63 (− 12.73, − 6.53)
Depression	27	97.63 (19.09)	6038	105.13 (16.28)	− 7.50 (− 13.67, − 1.34)	− 5.09 (− 11.58, 1.39)
PDD	10	82.00 (18.48)	6055	105.14 (16.28)	− 23.14 (− 33.24, − 13.04)	− 24.47 (− 35.68, − 13.27)

a Adjusted for age at IQ testing, sex, ethnicity, maternal social class and highest education.

**Table 3:** ORs for PEs at 13 years for psychiatric disorders at 8 years.

Psychiatric disorder at 8 years	Total No.	PEs, No. (%)	OR (95% CI) for PEs at 13 years		
Unadjusted	Model 1^a^	Model 3^b^
Any disorder	5528	673 (13.02)	1.76 (1.35–2.30)	1.82 (1.39–2.38)	1.96 (1.47–2.68)
Oppositional/conduct	5519	710 (13.25)	1.70 (1.15–2.52)	1.78 (1.20–2.64)	1.81 (1.17–2.80)
Anxiety	5528	717 (13.35)	1.60 (1.07–2.38)	1.62 (1.08–2.41)	1.70 (1.10–2.61)
ADHD	5519	723 (13.35)	1.55 (0.94–2.53)	1.65 (1.00–2.72)	1.91 (1.22–3.26)
Depression	5528	741 (13.46)	3.22 (1.29–7.99)	3.21 (1.29–7.99)	3.00 (1.13–7.96)
PDD	5528	743 (13.46)	8.03 (2.15–29.99)	8.31 (2.22–31.05)	11.79 (2.77–50.13)

a Adjusted for sex.

b Adjusted for age at PEs, sex, maternal social class and highest education.

**Table 4:** Mediation of the psychiatric disorder-PEs relationship by IQ.

Mediator	OR (95% CI) for PEs
IQ at 8 years	
Controlled direct effect^a^	1.58 (1.13–2.22)
Natural direct effect^b^	1.05 (1.01–1.10)
Total effect^c^	1.66 (1.19–2.33)

a The effect of any psychiatric disorder (exposure) on the risk of PEs (outcome) after adjusting for IQ (mediator).

b The effect on the risk of PEs of a change in IQ among unexposed individuals compared with exposed individuals.

c The product of controlled direct effect and natural direct effect.
